# Supplementary material for: Semantic composition in experimental and naturalistic paradigms
Source: Imaging Neurosci (Camb). 2024 Jan 22;2:imag-2-00072. doi: 10.1162/imag_a_00072 (PMC12224452; doi:10.1162/imag_a_00072)
Supplement: Supplementary Material [file imag_a_00072-supp.pdf]

Supplementary material

We used the entire epoch for computing the channel noise covariance matrix for the naturalistic data. However, we only used the 100 ms intervals prior to each trial as the baseline for the two-word data, aligning with previous literature on phrasal composition in a two-word setting (Bemis & Pylkkanen, 2011; Li & Pylkkänen, 2021). Since this may lead to different signal-to-noise ratios (SNRs) for the noise covariance estimates for the two-word and the naturalistic data, we reprocessed the two-word data, based-lined corrected using the whole epoch. The results are very similar to our initial findings (see Supplementary Figure 1).

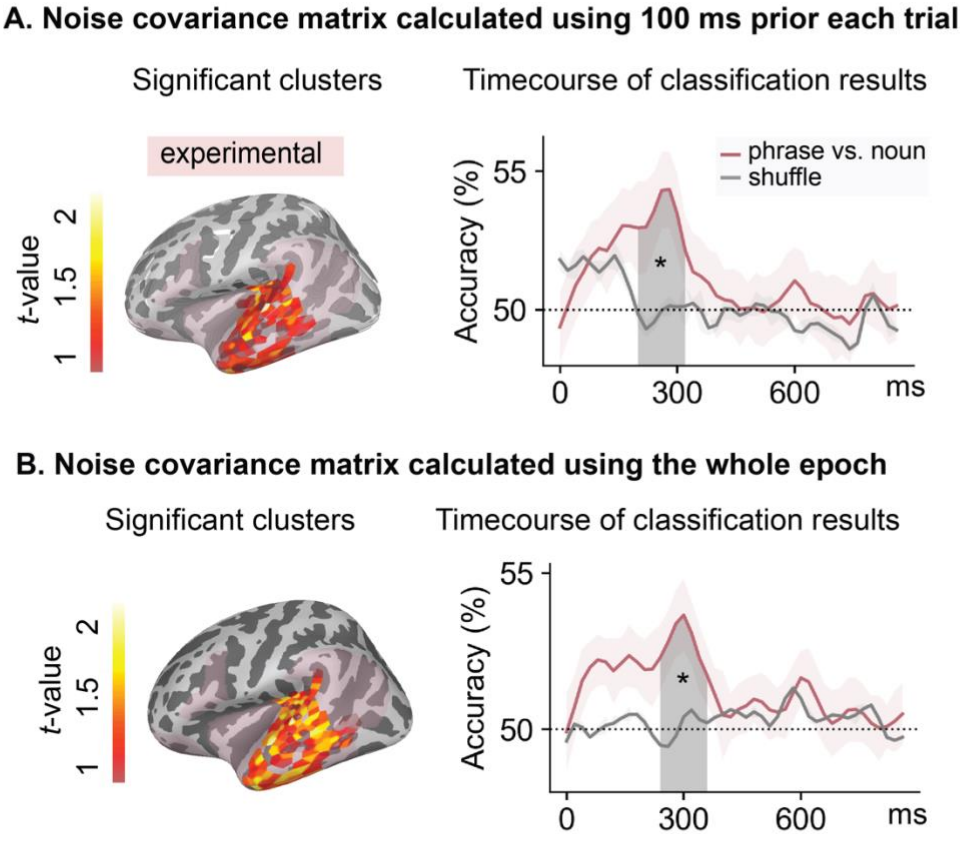

Supplementary Figure 1. The classification results of the two-word data. **A.** Noise covariance matrix calculated using the 100 ms before each trial. The classifiers can distinguish phrases from single nouns in the left anterior and middle temporal lobe from 200-340 ms (p=0.005) after the onset of the word. **B.** Noise covariance matrix calculated using the whole epoch. The

classifiers can distinguish phrases from single nouns in the left anterior and middle temporal lobe from 240-380 ms (p=0.007) after the onset of the word.
